# Factors influencing microgame adoption among secondary school mathematics teachers supported by structural equation modelling-based research

**DOI:** 10.3389/fpsyg.2022.952549

**Published:** 2022-09-08

**Authors:** Tommy Tanu Wijaya, Yiming Cao, Martin Bernard, Imam Fitri Rahmadi, Zsolt Lavicza, Herman Dwi Surjono

**Affiliations:** ^1^School of Mathematical Sciences, Beijing Normal University, Beijing, China; ^2^Pendidikan Matematika, Universitas Negeri Yogyakarta, Yogyakarta, Indonesia; ^3^Fakultas Keguruan dan Ilmu Pendidikan, Universitas Pamulang, Tangerang, Indonesia; ^4^Linz School of Education, Johannes Kepler University Linz, Linz, Austria

**Keywords:** learning media, technology acceptance model, TAM, microgame, structural equation modelling (PLS-SEM)

## Abstract

Microgames are rapidly gaining increased attention and are highly being considered because of the technology-based media that enhances students’ learning interests and educational activities. Therefore, this study aims to develop a new construct through confirmatory factor analysis, to comprehensively understand the factors influencing the use of microgames in mathematics class. Participants of the study were the secondary school teachers in West Java, Indonesia, which had a 1-year training in microgames development. We applied a quantitative approach to collect the data *via* online questionnaires through google form. Structural Equation Modelling (SEM) with AMOS software was used to analyze the proposed model. Empirical results confirmed the perceived easy to use and subjective norm influence (PEU and SN) relationship with teachers’ microgame usage behaviors and intentions. In this condition, SN was found to have the initial significant influence on behavioral intention (BI), as attitude, BI, and facilitating conditions also correlated with the actual use of microgames. Furthermore, the largest influential factor was BI, with the results subsequently showing that TPACK had no significant influence on the actual use of microgames. This report is expected to help bridge the gap across several previous studies, as well as contribute to the explanation and prediction of the factors influencing the teachers’ mathematical utilization of the study’s program. Besides this, it also helps to increase the use of microgames in teaching and learning activities.

## Introduction

Microgames are easily used by teachers as one of the numerous technology-based learning media, to facilitate more students’ interests in the classroom (Grace et al., 2015; Rahmadi et al., 2021). This is based on having a positive effect at all education levels, such as learning outcome improvements, educational interest, and teacher-student interactions (Brom et al., 2011; Chai-Arayalert and Puttinaovarat, 2021). According to Chai-Arayalert and Puttinaovarat (2021), students were happier when the teacher provided microgames to help them understand the lesson materials, leading to an interesting and eventful learning process. The use of this game has also been widely examined (Brom et al., 2011; Açıkgül and Aslaner, 2020; Rahmadi et al., 2022), although the factors influencing its utilization by teachers have not been highly evaluated. This indicates that a related report is very important in confirming these various influential factors, accompanied by the initiatives to disseminate the use of the game and appropriately train teachers. Besides focusing on entertainment, Serious Microgames are also created to transfer new knowledge points to students (Moffat and Shabalina, 2017; Kulanthaivel and Ulagamuthalvi, 2020; López et al., 2021). In this condition, the games aim to convey and improve the students’ mathematical knowledge points and, abilities, as well as change their attitudes and behaviors towards the assumption that mathematics is a difficult and boring subject (Moshell et al., 2007; Garneli et al., 2019; Mayfield et al., 2019). They are also one of the numerous digital media combined with game-based learning methods, to increase mathematical performance expectancy (Molin, 2017). Subsequently, these games are observed to facilitate and provide a new perspective on mathematics teaching and learning activities, towards the increase of students’ motivation, creativity, and emotionality (Meletiou-Mavrotheris and Prodromou, 2016; Chadli et al., 2019; Rahmadi and Lavicza, 2021). This method enables great students’ activeness during learning activities and allows them to achieve higher performance than the traditional models without digital media (Pistoljevic and Hulusic, 2017; López et al., 2021). It also improves students’ mathematical problem-solving abilities (Malvasi and Gil-quintana, 2022), based on the development of solutions for arithmetical issues.

Despite these benefits, the prediction of the factors influencing mathematics teachers’ intention and actual use of microgames is still needed. This is very important for increasing the use of these microgames. Therefore, this study aims to carry out the following:

1. To predict and identify factors influencing mathematics teachers’ behavioral intentions and actual use of microgames.
2. To predict and identify the largest positive significant factor affecting these teachers’ behavioral intentions and actual uses of microgames.

To achieve these goals, a technology acceptance model Davis (1989) and initial hypothesis were used and developed, respectively. The relationship between the influential factors was also tested using a structural equation model (SEM) approach Hair et al. (2016). This paper is subsequently divided into the following parts, (a); Section “Introduction”, where the introduction explains the background of the problem, study objectives, and novelty, (b) Section “Literature review”, where the literature review explains the concept of microgames, teachers’ acceptance model, and the initial hypothesis, (c) Section “Study Methodology”, where the study sample, as well as the data collection steps and analysis, are comprehensively explained, (d) Section “Data Analysis and Results”, which presents the results of the data normality test, as well as the measurement and structural model analyses, (e) Section “Discussion”, where the discussion and study implications are presented, and (f) Section “Implications”, where the conclusion is drafted based on the results.

## Literature review

### Microgames with visual basic for application

Visual Basic for Applications (VBA) is a Microsoft programming code intentionally provided to users as an object-oriented language, leading to the free improvement of performances according to their needs (Esawi, 2009; Kareth et al., 2018). In education, this program is widely used by teachers to develop interesting learning media, due to being familiar without the requirement of additional platforms (Molin, 2017; Bernard and Chotimah, 2018; Açıkgül and Aslaner, 2020; Rohaeti et al., 2020). As a programming language, it also has a simple structure, which is easily understood and modified by teachers (Orosa García, 2011). This confirms that VBA users are able to design and extend Microsoft office functions into interesting learning media and microgames. In some previous studies, this program was widely developed as a technology-based learning media and microgames (Novita et al., 2016; Pedrami et al., 2016; Kareth et al., 2018; Rohaeti et al., 2019). According to Arifin and Yeop (2012), the use of Visual Basic in MS Excel was easier and more interesting than that of other Microsoft programs, although many of them were programmatically supported. In Taiwan, Excel VBA is known as a powerful tool in nurse education, causing many universities to subsequently open special courses to teach prospective medical personnel about the utilization pattern of the program (Lee et al., 2014).

**Figure 1:**
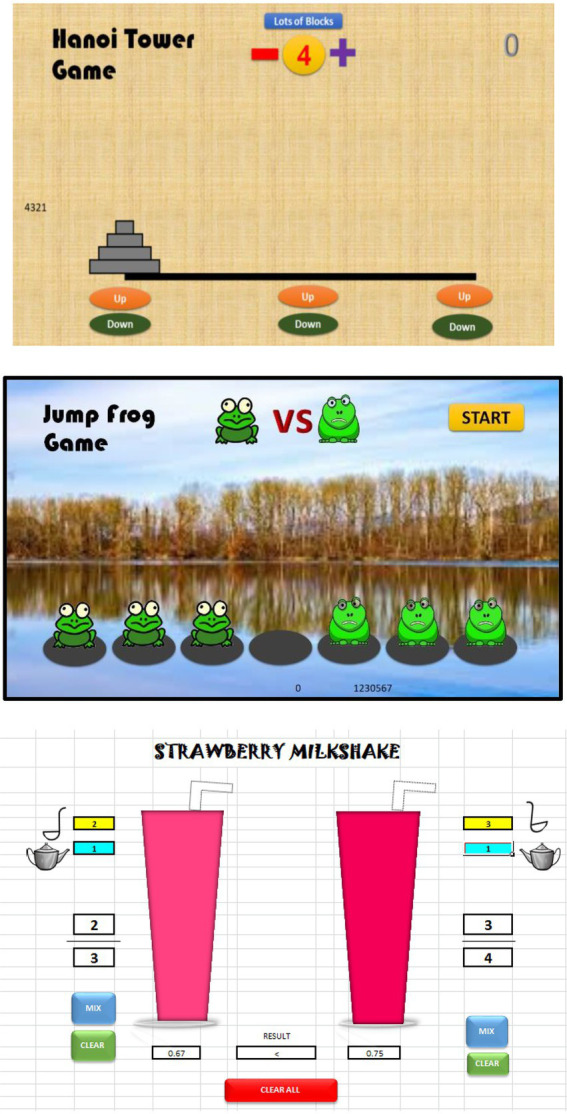
Examples of user-generated microgames with VBA Microsoft office.

This report used microgames with Microsoft Office VBA, a media content created by users, for example, the mathematics teacher (Murni et al., 2020; Rahmadi and Lavicza, 2021). Although the teachers are not professional developers, these games are still more suited to the students’ curriculum, learning objectives, and characteristics when teaching mathematics. The texts, sounds, images, interesting animation, and videos, the content is often developed personally by the teachers or with the free acquisitions obtained from many sources. Moreover, these advantages facilitate teachers’ modification, distribution, and utilization during free teaching. Students are found to also internally or externally use the microgames provided during learning processes, through blogs, email, WhatsApp, or other platforms. This is because of the light file extension, as some examples of these Excel VBA games include the following:

1. The Tower of Hanoi (The problem of Benares Temple or Tower of Brahma) is a math game or puzzle containing three pegs with at least three rings (Lam, 2006; Alexander et al., 2016). In this condition, each ring has a different size and is stacked in one ascending capacity, for instance, the smaller ones lay atop the larger types. This subsequently shows that the number of rings is often increased, although the towers remain 3. The Tower of Hanoi is often solved using the formula, **2**^**n**^**−1**, leading to the three rings being calculated as **2**^**3**^**–1 = 7**. This game has various learning objectives for improving students’ logic and thinking stages, which are highly required by computer scientists, mathematicians, and engineers. It is also commonly suitable for students in grades 9–12, where logical thinking skills are quite good.
2. The Frog Jumping problem is a simple game of sequences and algorithms, which improves computational thinking and mathematical problem-solving abilities (Xiong and Lee, 2007). In this condition, students are instructed to move the frog from right to left with some rules, as the pattern towards goal achievement is observed as $n^{2}+2n=X$ , where *n* and X = the number and jumps of the frogs, respectively. This is often suitable for junior and senior high school levels, where students already have good abstract thinking skills.
3. The math of a milkshake is a microgame used at the elementary school level, where fractions are being studied. These are associated with a contextual problem, where milkshakes are the preferred drink for children. Firstly, the students are instructed to prepare two favorite drinks, with options ranging from a strawberry and blueberry milkshake, as well as a Thai and green tea latte. This was accompanied by the mixture of powder and water, whose application ratios were subsequently observed in fractions and decimals. Finally, the students were able to observe the most dominant drink color, which exhibited a greater fraction. These Excel VBA games are shown in Figure 1, as teachers were found to subsequently explain the concept of fractions after the learning media simulation observation. They also evaluate the relationship between normal and decimals fractions, respectively.

Based on Figure 1, the composing method was studied by the study respondents, as these games were observed to improve mathematics learning outcomes according to previous reports (Wijaya et al., 2020). After understanding the exact influence of microgames on students’ abilities, an analysis of teachers’ utilization intentions was one of the initial steps to be performed, indicating that the use of these games in teaching and learning activities had a maximum effect.

### Development of the research model and initial hypotheses

#### Technology acceptance model

Technology acceptance model is the first model to examine the psychological factors influencing an individual to accept new technology (Davis, 1989; Al-maroof and Al-emran, 2018). It also predicts and explains user behavioral intention towards the utilization of various technology-based media (Tan, 2013; Briz-Ponce et al., 2017). Furthermore, the relationship between perceived benefits and ease of use, as well as user attitudes and behavioral intentions to utilize new technologies, is the construct within the TAM model (Bando et al., 2017; Al-Rahmi et al., 2021a,b). This predicts that the behavioral intention is the main factor determining the users’ actual utilization of the new technology. In TAM, the relationships observed between behavioral intention and actual use have reportedly been verified by many studies, such as Al-Rahimi et al. (2013), Abdul Rabu et al. (2019), and Alismaiel (2021). Despite being widely used to predict user intentions in all scientific fields, the TAM model still had some limitations. According to Dishaw and Strong (1999), this model was popularly used in the business field, leading to the need to examine it in other science sectors. This was based on exploring and validating the suitability of TAM, for predicting the users’ utilization intention of the new technology within other fields, including in education (Legris et al., 2003) also stated that the study should be flexible and include external variables in the TAM model. External indicator adalah indicator tambahan yang ditambahkan pada technology acceptance model yang diprediksi dapat menjelaskan behavioral intention dan usage beahvior of microgames by mathematics teachers. This was to predict and explain the users’ behavioral intentions and actual use of new technologies. These were in line with previous studies, where this model was recently modified to predict the BI and AU of new technology, such as TPB, UTAUT, and TTF (Hanham et al., 2021; Jiang M. Y. et al., 2021; Al-bashayreh et al., 2022).

By modifying the Technology Acceptance Model (Davis, 1989), the proposed model of this present report was developed, with the addition of some factors, namely self-efficacy, subjective norm, facilitating conditions, and TPACK. This was to predict and explain the significant factors influencing Indonesian teachers towards using microgames in mathematics. Based on (Anthony et al., 2021), a strong relationship was also observed between UTAUT and TPACK. Meanwhile, TPACK and TAM were simultaneously used to predict the use of new technology. Despite this, no studies were found on the integration of TAM and TPACK, in investigating the use of microgames. This revealed that the present report adopted the TPACK to the TAM model, with UTAUT subsequently suspected as one of the influential factors in the actual usage of microgames by the math teachers.

##### Perceived usefulness

Perceived usefulness is the degree to which a user believes that the use of technology helps to improve work performance (Davis, 1989). In this present report, it was defined as the pattern by which the mathematics teachers assumed that microgames helped them to teach more effectively and create a fun class. Based on these conditions, several previous studies clarified that PU had a significant effect on teacher attitudes and motivated them to use technology-based learning media (Wang, 2015; Osih et al., 2020; Drueke et al., 2021). It also had a direct effect on the educational behavioral intention and attitude towards ICT, with more studies tagging it as the biggest significant factor influencing users’ BI in using new technology (Camadan et al., 2018; Sukendro et al., 2020; Fussell and Truong, 2021). Therefore, the proposed hypotheses are as follows:

*H1*: PU has an effect on the attitude of mathematics teachers towards using microgames.

*H2*: PU has an effect on the behavioral intention of mathematics teachers towards using microgames.

##### Perceived ease of use

Perceived ease of use is defined as the degree to which a person assumes that new technology does not require much effort (Venkatesh et al., 2003). This indicates that a person is more willing to utilize technology when it is easy to use (Al-Rahmi et al., 2021a,b; Jiang H. et al., 2021). However, in this present report, it was defined as the level where mathematics teachers assumed that microgames were easy to use during teaching and learning activities. Based on previous studies, PEU had a relationship with attitudes toward ICT and the behavioral intention of users (Hwang et al., 2014; Ansong-Gyimah, 2020; Gurer and Akkaya, 2021). It also had a significantly positive and direct effect on the use of new technology (Arpaci et al., 2020; Al-Rahmi et al., 2021a,b; Huang, 2021). Therefore, the proposed hypotheses are as follows:

*H3*: PEU has an effect on the attitude of mathematics teachers towards using microgames.

*H4*: PEU has an effect on the behavioral intention of mathematics teachers towards using microgames.

##### Teacher attitudes towards microgames

Based on some literature, the attitude of mathematics teachers to ICT was influenced by its usefulness and ease of use (Cheong et al., 2018; Agustyaningrum et al., 2021). In the original TAM model, user attitude towards ICT was influenced by PU and PEU (Gellerstedt et al., 2018), indicating its effects on the actual use of technology-based learning media. In this present study, attitude is found to describe the behavior of mathematics teachers toward microgames, as a medium helping to improve student mathematical learning performance and effectiveness. Therefore, the proposed hypothesis is as follows:

*H9*: teachers’ attitudes affect the actual use of microgames.

#### External factors

External indicators are additional indicators added to the technology acceptance model which are predicted to explain behavioral intention and usage behavior of microgames by mathematics teachers. To specifically and comprehensively predict and explain the BI and AU of microgames by mathematics teachers, the proposed model was modified by adding the following four variables to the TAM model construct.

##### Subjective norm

Subjective norm is defined as the degree to which a person positively responds to the surrounding perceptions responsible for the influence of a behavior (Davis, 1989). Several studies showed that subjective norms had a relationship with BI (Courtois et al., 2014; Saleem et al., 2021; Stössel et al., 2021), which also affected a person’s AU to use new technology. Therefore, the proposed hypothesis is as follows:

*H5*: Subjective norm affects mathematics teachers’ behavioral intention to use microgames.

##### Facilitating condition

This is the technical and organizational infrastructure support needed to use the new technology (Venkatesh et al., 2012). In this study, it was observed as the teacher support and computer equipment needed to use microgames during mathematical teaching processes. FC is also observed as a factor of AU, regarding the use of institutional technology-based learning media (Suki and Suki, 2017; Liebenberg et al., 2018). This explains that it is expected to affect mathematics teachers’ actual use of microgames, during teaching and learning activities. Therefore, the proposed hypothesis is as follows:

*H6*: Facilitating conditions affects mathematics teachers’ behavioral intention to use microgames.

##### Self-efficacy

Self-efficacy is the degree to which people believe in their abilities to conduct effective and life-changing performances (Compeau and Higgins, 1995; Lavidas et al., 2022). Meanwhile, in this report, Self-efficacy is defined as technological self-efficacy, where the mathematics teachers believe in their abilities to use and integrate microgames during mathematics education. Based on previous literature, SE was predicted to have a relationship with behavioral intention (Lavidas et al., 2022), with a strong correlation observed with a person’s attitude towards new technology. Therefore, the proposed hypotheses are as follows:

*H7*: Self-efficacy affects mathematics teachers’ attitudes toward using microgames.

*H8*: Self-efficacy affects mathematics teachers’ behavioral intention towards using microgames.

##### Technological pedagogical content knowledge

**Figure 2:**
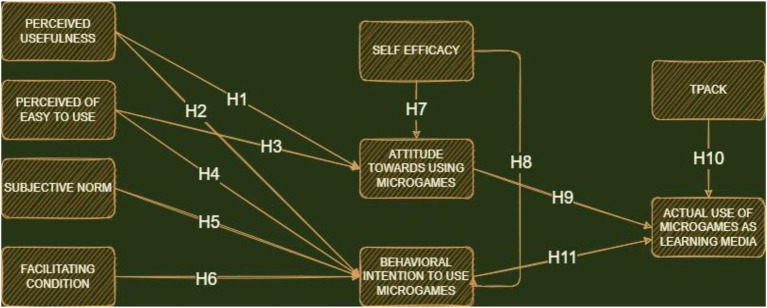
Initial hypothesis and model development.

Technological pedagogical content knowledge was developed from the work of Mishra and Koehler (2006), as a proposed model integrating technology into educational activities, to achieve effective learning. According to previous reports, this model had a relationship with the actual use of technology in teacher classrooms (Anthony et al., 2021). This explained that people were willing to use new technology when they have TPACK skills, due to their understanding in the integration patterns of the technical teaching and learning media in the classroom. It is also the original model adopted by many scientific fields (Cheung et al., 2018), which is still being actively, conceptually, theoretically, and empirically developed and modified by scholars and lecturers. Several previous studies also suggested and proved that TPACK had a relationship with the technology acceptance model (actual use; Wah and Hashim, 2021). Microgames are not similar to several technology-based mathematics learning media, such as arithmetical dynamic software or micro-lectures which solely emphasizes teacher control. This indicates that microgames are used by students, with teachers required to understand and master appropriate integration approaches and learning models. These conditions lead to the initial hypothesis which stated that the teachers’ TPACK ability has a relationship with the actual use of microgames (Figure 2). Despite the results, this present report still aims to analyze whether TPACK has a relationship with mathematics teachers’ actual usage of microgames. Therefore, the proposed hypothesis is as follows:

*H10*: TPACK affects the actual uses of microgames similarly as a learning media.

## Study methodology

This study was carried out in three stages, (1) a questionnaire was adopted from the TAM of previous studies and designed for data collection processes, (2) the SEM model was used to examine the initial hypothesis of the factors influencing the teachers’ behavioral intention and actual use of microgames, before the presentation of the sample characteristics, and (3) the data analysis process was described to confirm the data evidence.

### Instrument

A total of nine measurement tests were designed on a structural model with 27 questionnaire items. The collection of data on students’ microgame perceptions also used a questionnaire with a 5-point Likert scale (Rahayu et al., 2020), from a range of 1–5 for SD-SA (strongly disagree-strongly agree). Additionally, each construct had 2–4 measurement items, with the details of the nine variables and 27 questions observed in Supplementary Appendix 1. All the proposed items were also supported by a literature review.

### Participants and data collection procedures

Since 2018, training has been observed on the development of microgames in Bandung, West Java, Indonesia. This is carried out by using VBA, Microsoft Excel, and PowerPoint, towards introducing technology-based learning media to the provincial secondary mathematics teachers. It also aims to improve the quality and integration of mathematics learning and ICT in the classroom, respectively. Furthermore, it is expected to change the perspective of students regarding the difficulties and boredom of mathematics, during the teacher’s utilization of microgames as a learning media. In this report, questionnaires were randomly distributed to teachers with experience in mathematical game development, based on the collection of data. To meet ethical requirements, an ethical approval was obtained from the Faculty of Mathematics and Science Education, IKIP Siliwangi, Bandung, West Java. In this condition, voluntary participation was adopted, in which participants were allowed to withdraw at any time when they have comfortably answered the questionnaire items. Subsequently, 4 of the 194 data were deleted due to being incomplete, indicating that a total of 190 pieces of information were declared valid. Table 1 shows that 38.9% and 61.1% of the respondents are male and female, with 74.2% and 25.8% being bachelor’s and master’s degree holders, respectively.

**Table 1:** Data demographics respondent.

Basic information	description	*N*	percentage
gender	Male	74	38.9%
	Female	116	61.1%
Level education	S1	141	74.2%
	S2	49	25.8%
Age	20–25	91	47.89%
	26–30	71	37.37%
	31–35	16	0.08%
	Above 36	12	0.06%
Type of school	Public school	151	79.47%
	Private school	39	20.53%

### Data analysis

Using the extension of TAM, the PEU, PU, SN, FC, TA, SE, TPACK, BI, and AU (Perceived Ease of use, Perceived Usefulness, Subjective Norm, Facilitating Condition, Teachers Attitude, Self-Efficacy, Technological Pedagogical Content Knowledge, Behavioral Intention, and Actual Use of microgames) were measured. Moreover, the procedural steps from previous studies were adequately applied to this experiment, whose data were analyzed using the PLS-SEM technique developed and redeveloped by Huang et al. (2020) and several scholars, including Hu and Bentler (1998), Dijkstra (2010), as well as Henseler et al. (2015). In this condition, the technique subsequently applied OLS (ordinary least squares) regression to predict the path model’s partial relationship, towards maximizing and minimizing the explained variance and errors in endogenous constructs, respectively (Hair et al., 2016). Using smart-PLS 3.0, the data normality test was confirmed in the first stage, with the validity and reliability of the measurement model being implemented in the second phase. Construct validity was also determined by analyzing the loading factors, Cronbach’s alpha, and convergence viability, before entering the structural model and hypothetical testing stages.

## Data analysis and results

This section is divided into several categories, i.e., (1) the data normality analysis, (2) the measurement model analysis, and (3) the structural model assessment. Using PLS-SEM, the systematic evaluation steps were suggested by Hair et al. (2016).

### Normality test

Before the measurement and structural model analyses, the normality test on each variable item was initially carried out, with the data being pronounced normal when the kurtosis and skewness values are between |10| and |3|, respectively (Moorthy et al., 2019). This clarified that the data had kurtosis and skewness values between −1.307 to 3.627 and −0.885 to 0.325, respectively, as shown in Table 2. Based on these results, all the available data were fairly normally distributed.

**Table 2:** Descriptive statistics and normality testing.

No.	construct	Mean	Median	Min	Max	Standard deviation	Excess kurtosis	Skewness
1.	PU1	4.611	5.000	3.000	5.000	0.529	−0.369	−0.883
2.	PU2	4.421	4.000	3.000	5.000	0.554	−0.914	−0.245
3.	PU3	4.316	4.000	3.000	5.000	0.669	−0.762	−0.470
4	PEU1	3.768	4.000	1.000	5.000	0.852	0.607	−0.772
5.	PEU2	4.074	4.000	2.000	5.000	0.757	0.560	−0.710
6.	PEU3	3.926	4.000	2.000	5.000	0.811	−0.452	−0.341
7.	SN1	3.084	3.000	1.000	5.000	0.866	0.216	0.325
8.	SN2	3.189	3.000	1.000	5.000	0.932	−0.168	−0.230
9.	FC1	3.411	3.000	1.000	5.000	0.827	0.405	−0.330
10.	FC2	4.021	4.000	1.000	5.000	0.808	1.361	−0.885
11.	FC3	3.905	4.000	1.000	5.000	0.859	0.578	−0.719
12.	TA1	4.547	5.000	3.000	5.000	0.518	−1.307	−0.420
13.	TA2	4.305	4.000	3.000	5.000	0.617	−0.639	−0.308
14.	TA3	4.537	5.000	3.000	5.000	0.539	−0.885	−0.554
15.	TA4	4.389	4.000	3.000	5.000	0.529	−1.101	0.023
16.	SE1	4.158	4.000	1.000	5.000	0.701	0.627	−0.971
17.	SE2	4.105	4.000	2.000	5.000	0.640	0.356	−0.340
18.	SE3	4.263	4.000	1.000	5.000	0.668	2.956	−1.217
19.	TPACK1	4.084	4.000	2.000	5.000	0.777	0.331	−0.691
20.	TPACK2	4.032	4.000	2.000	5.000	0.732	0.790	−0.699
21.	TPACK3	3.979	4.000	1.000	5.000	0.833	1.290	−0.951
22.	BI1	3.495	4.000	2.000	5.000	0.752	−0.317	−0.057
23.	BI2	3.789	4.000	2.000	5.000	0.694	0.380	−0.450
24.	BI3	3.663	4.000	2.000	5.000	0.776	−0.220	−0.285
25.	UB1	3.274	3.000	1.000	5.000	0.956	−0.565	−0.064
26.	UB2	4.095	4.000	2.000	5.000	0.697	1.025	−0.696
27.	UB3	3.358	3.000	1.000	5.000	0.951	−0.508	−0.253

### Measurement model test

The validity test of the measurement model was observed in the content, convergent, and discriminant validities (Cheng and Tsai, 2020; Habibi et al., 2020). This indicated that the measured items were adopted from the existing literature, with an initial investigation subsequently carried out. Based on the results, the content validity of the model was quite good. Furthermore, Table 3 showed that the loading factors between each measured and latent variable were more significant than the correlation coefficient with other determinants (cross-factor loading). This proved that the measurement model analysis had good convergent and discriminant validities. In Table 3, the Cronbach alpha, Construct reliability, and average variance extracted (AVE) were also observed. Convergent validity is described as a condition for relating to the variable construct. This was declared ideal and good when the AVE was more than 0.5 (Hair et al., 2016). All the measurement models observed also have good convergence validities, with the lowest AVE value of attitude being 0.635. Moreover, ICR (Internal Consistency Reliability) was implemented to evaluate the consistency of results across all indicators, where the value of Cr and CA (Composite reliability and Cronbach Alpha) should be 0–1 (Hair et al., 2016). In this condition, the model reliability was said to be good when the CR and CA were not <0.7.

**Table 3:** Factor loadings, VIF, reliability and validity statistics.

Construct	VIF	ATT	BI	EE	FC	PU	SE	SN	TPACK	UB	VIF	Cronbach A	CR	AVE
TEACHERS ATTITUDE												0.809	0.874	0.635
TA1	1.685	0.751									1.685			
TA2	2.104	0.852									2.104			
TA3	1.785	0.788									1.785			
TA4	1.851	0.793									1.851			
BEHAVIORAL INTENTION												0.838	0.903	0.755
BI1	1.978		0.874								1.978			
BI2	1.912		0.858								1.912			
BI3	2.009		0.875								2.009			
Perceived Easy to Use												0.848	0.908	0.767
PEU1	1.902			0.842							1.902			
PEU2	2.067			0.884							2.067			
PEU3	2.361			0.900							2.361			
FACILITATING CONDITION												0.720	0.836	0.630
FC1	1.199				0.786						1.199			
FC2	1.772				0.816						1.772			
FC3	1.867				0.778						1.867			
PERCEIVED USEFULLNESS												0.791	0.878	0.706
PU1	2.002					0.891					2.002			
PU2	1.588					0.821					1.588			
PU3	1.649					0.806					1.649			
SELF EFFICACY												0.763	0.863	0.678
SE1	1.531						0.816				1.531			
SE2	1.793						0.878				1.793			
SE3	1.475						0.774				1.475			
SUBJECTIVE NORM												0.764	0.895	0.809
SN1	1.620							0.892			1.620			
SN2	1.620							0.907			1.620			
TPACK												0.894	0.934	0.826
TPACK1	1.958								0.849		1.958			
TPACK2	4.090								0.929		4.090			
TPACK3	4.340								0.946		4.340			
ACTUAL USE												0.760	0.863	0.677
AU1	1.738									0.838	1.738			
AU2	1.341									0.764	1.341			
AU3	1.816									0.864	1.816			

Table 4 shows that the entire AVE square root of the latent variable is larger than the correlation coefficient of other determinants, verifying that the discriminant validity of this analysis is good (Fornell and Larcker, 1981). Besides considering the Fornell–Larcker test (1981), Hair et al. (2006) also proposed to observe the value of HTMT (heterotrait–monotrait ratio of correlations), to highly analyze discriminant validity specifically. In this approach, DV is considered good when the HTMT value does not exceed the 0.9 threshold. Using smart-PLS, Table 5 also reveals that the highest HTMT value is 0.829 (PU-TA), proving that the DV between the latent variables is good.

**Table 4:** Fornell–Larcker test for discriminant validity test.

	TA	BI	FC	PEU	PU	SE	SN	TPACK	AU
TA	**0.797**								
BI	0.442	**0.869**							
FC	0.262	0.293	**0.794**						
PEU	0.554	0.537	0.335	**0.876**					
PU	0.667	0.314	0.159	0.379	**0.840**				
SE	0.619	0.327	0.244	0.457	0.447	**0.824**			
SN	0.085	0.402	0.215	0.301	0.049	0.221	**0.900**		
TPACK	0.592	0.564	0.226	0.569	0.442	0.451	0.305	**0.909**	
AU	0.476	0.643	0.334	0.523	0.277	0.399	0.314	0.451	**0.823**

All bolded loadings in the diagonal dimension are larger than those in the vertical.

**Table 5:** HTMT (heterotrait–monotrait ratio of correlations) values.

	TA	BI	FC	PEU	PU	SE	SN	TPACK	AU
TA									
BI	0.531								
FC	0.353	0.356							
PEU	0.645	0.640	0.415						
PU	0.829	0.387	0.273	0.457					
SE	0.793	0.400	0.346	0.570	0.574				
SN	0.165	0.499	0.245	0.382	0.164	0.283			
TPACK	0.684	0.651	0.301	0.649	0.536	0.549	0.367		
AU	0.604	0.801	0.415	0.649	0.358	0.524	0.410	0.547	

### Structural model

Before the structural model analysis, the values of VIF (Variance Inflation Factor) need to be evaluated to analyze collinearity between indicators. To prove the unbiased nature of the model, the VIF value should not be more than 5 (Hair et al., 2016). Based on Table 3, the largest and average VIF values were 4.34 and <2.00, respectively. This confirmed that the collinearity between constructs did not have a critical problem, proving that the initial model was not distorted or inaccurate. From these results, the utilized model had no bias problem, with the values of RMS_theta, NFI, and standardized root mean square residual (SRMR) being commonly used as PLS-SEM indicators, to evaluate the appropriateness of the overall technique.

**Figure 3:**
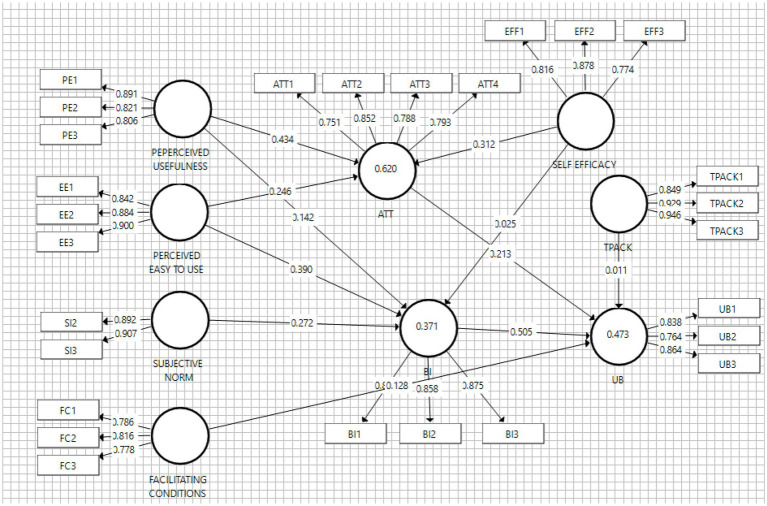
The structural model with factor loading and squared multiple correlations.

The SRMR value is observed from 0 to 1 and pronounced a good fit model when <1.00 (Hu and Bentler, 1998). Therefore, the results showed that a value of 0.077 was observed for this model, indicating a good fit. Greater NFI values above 0.9 are also found to be a good fit for better performances (Hu and Bentler, 1998). Despite this value being 0.885 and <0.9, it was still within the acceptable range (Huang, 2021). RMS-theta value is only suitable for evaluating reflective measurement models, with a value <0.12 indicating adequate model fitness (Henseler et al., 2015). Since the RMS_theta value of this analysis was 0.113, the model was generally and reasonably well-fitted. In assessing the structural model, the path relationship, effect size, R^2^, and predictive relevance model need to be analyzed, based on the recommendations from Hair et al. (2016). To analyze this model and validate the hypothesis, Smart-PLS 3 was used by bootstrapping 5,000 samples towards examining the significance of the path coefficients. The results are subsequently shown in Figure 3, regarding the PLS-SEM factors influencing the microgame utilization of mathematics teachers.

**Figure 4:**
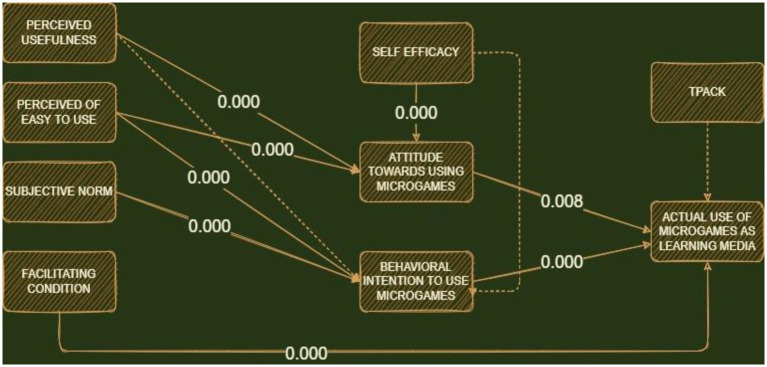
Final model with *p*-value, the dotted line shows the path is not significant.

In path analysis, the *t*-value was used to test each hypothesis, where a significant level of 0.05 was obtained when the *t*-value was >1.96. Meanwhile, significant levels of 0.01 and 0.001 were also observed when *t*-value >2.58 and 3.29, respectively. Table 6 shows that H11, H3, H4, H1, H7, and H5 achieved a significance level where the *p*-value was <0.001. In this condition, H6 and H9 also reached a significant *p*-value of 0.05, while H2, H8, and H10 were insignificant for being more than 0.05. The structural and final models with loading factor and *p*-value are subsequently shown in Figures 3, 4, respectively.

**Table 6:** Summary of hypothesis testing results.

Hypothesis		Path coefficient (*β*)	Mean (*M*)	STDEV	*T* statistics	Values of *p*	Result
H1	PU → TA	0.434	0.432	0.054	8.099	0.000	Significant
H2	PU → BI	0.142	0.139	0.074	1.924	0.055	Not significant
H3	PEU → TA	0.246	0.241	0.052	4.724	0.000	Significant
H4	PEU → BI	0.390	0.389	0.068	5.704	0.000	Significant
H5	SN → BI	0.272	0.271	0.058	4.706	0.000	Significant
H6	FC → AU	0.128	0.139	0.050	2.569	0.010	Significant
H7	SE → TA	0.312	0.322	0.065	4.798	0.000	Significant
H8	SE → BI	0.025	0.028	0.089	0.284	0.776	Not significant
H9	TA → AU	0.213	0.212	0.080	2.650	0.008	significant
H10	TPACK → AU	0.011	0.012	0.072	0.157	0.876	Not significant
H11	BI → AU	0.505	0.500	0.068	7.378	0.000	Significant
SRMR = 0.087							

Table 6 showed that the latent variable of the model did not have strong collinearity (VIF < 5), indicating the following (1) the items of the 7 constructs did not overlap, (2) each item independently reflected the indicators to be measured, and (3) the setting of the questionnaire was reasonable.

Furthermore, the *R*^2^ value (Table 7) is often used to describe the explainability of a model, with the value commonly observed between 0 and 1 (Shiferaw et al., 2021). This indicates that a higher *R*^2^ value led to greater explanatory power. Based on the results, 62%, 37.1%, and 47.3% of the TA, BI, and AU variances toward using microgames were explained through structural models.

**Table 7:** The squared multiple correlations *R*^2^.

	*R* square	*R* square adjusted
TA	0.620	0.614
BI	0.371	0.357
AU	0.473	0.462

## Discussion

This study aimed to develop and test the initial model, to predict the factors influencing the BI and AU of mathematics teachers towards using microgames during teaching. The attitude, TPACK, and self-efficacy were also integrated into the TAM, to evaluate these influential technology utilization determinants. In the empirical test, 8 of the 11 hypotheses were supported, with the determined PU, PEU, and SN becoming the crucial factors of teachers’ microgames BI during teaching and learning. Meanwhile, TA, BI, and FC were the crucial predictors of teachers’ AU, based on the utilization of the game. In this case, the behavioral intention of these teachers had the largest positive effect on actual use, indicating that the factor was highly considered when using the games. This revealed that greater intentions were an effective tool in increasing the teachers’ use of microgames during mathematics lessons. Furthermore, PEU had the greatest positive effect on the teachers’ behavioral intention to use these programs. This implies that the developers or government should highly consider the ease of using microgames to teach mathematical materials. These microgames are also utilized better when easily operated on multiple devices, including laptops, smartphones, and tablets, leading to the ease of use for both teachers and students during mathematics lessons. This was in line with many previous reviews, where PEU was the main factor influencing users to use new technology (Suki and Suki, 2017; García Botero et al., 2018; Abbad, 2021; Wijaya et al., 2022).

Subjective norms are the second most influential factor in the behavioral intention (BI) of mathematics teachers to use microgames. Based on the results, SN did not have a direct relationship with the actual new technology use, although significantly affected BI. This was in line with several previous studies, where the factor had a strong relationship with the use of ICT-based mathematics learning media (Han and Shin, 2016; Joa and Magsamen-Conrad, 2021). Furthermore, subjective norms emphasized the expectations of people around mathematics teachers, regarding the integration of microgames in educational activities. In this case, they assumed that people socially expected them to master the development of the learning model, due to having a strong desire to participate in campus or governmental trainings. From these results, the use of microgames was importantly considered by the mathematics teachers after the acquisition of various essential knowledge. For example, this learning model was considered an effective ICT-based media after the acquisition of developmental school trainings, to improve students’ mathematical outcomes. This proved that the support of the government for the use of microgames definitely affected the behavioral intention of mathematics teachers.

The attitude was the second biggest factor after the behavioral intention, regarding its significance to teachers’ actual uses of microgames. In Indonesia, attitude was expected to have a relationship with educational technology integration (Muhaimin et al., 2020). This supported the positive thinking of mathematics teachers towards the use of institutional technology. When teachers attitudes towards microgames was good, their use of the technology is also expected to be better. Based on the results, PU did not affect the teachers’ behavioral intention towards using the games in mathematics. Meanwhile, it was the main factor influencing users to use digital textbooks (Yoo and Roh, 2019). In contrast to other technology-based learning media, teachers observed that microgames were not often independently carried out, indicating that their teaching performances were not only influenced by microgames. In this condition, they may should be able to know the right combinative teaching patterns, to maximize the effects of the microgames towards producing fun classes, as well as increasing students’ interest and educational motivation. Compared to cognitive belief, the combination of easy-to-use games and supportive facilities was more likely to increase the teachers’ intention to use the programs during mathematics lessons. This is probably the reason PU did not significantly affect the behavioral intention of the teachers. Based on self-efficacy, a difference was observed for the teachers and students in Fianu et al. (2018), where SE was a factor influencing students to learn through MOOC. This showed that the factor did not affect the behavioral intention of the mathematics teachers. These results strengthened Muhaimin et al. (2020) and Teo et al. (2019), where self-efficacy had no significant relationship with the intention to use web-based learning. This proved that teachers’ SE was insufficient to influence their BI towards using microgames during mathematics lessons. In this case, greater efforts and other effective influential factors were required for the utilization of these games. In addition, Indonesian mathematics teachers believed that their abilities to use technology had no relationship with their usage intentions.

Facilitating conditions had a significant effect on the actual use of mathematics teachers, regarding the utilization of microgames. This explained that support systems and facilities acknowledged the integration of technology into teaching and learning activities. As a predictor influential to actual use, FC was believed to be importantly effective to users, towards using new technologies (microgames). Based on the Siliwangi Institute of Teacher Training and Education as well as Cimahi Technopark, a center was provided for learning and developing microgames, with the belief that teachers were capable of integrating microgames into their respective educational activities. In addition, the factor should not be lacking when examining the determinants influencing teachers’ intentions to use the games. Many scholars also assumed that teachers need to have knowledge skills to integrate technology into their classrooms (Schmidt et al., 2009; Chai et al., 2016; Wijaya and Hermita, 2021). This was not in line with some previous reports, where TPACK affected teachers’ preservice intentions to integrate technology upon entering the work field (Cheung et al., 2018; Anthony et al., 2021). In this condition, TPACK was not a predictor with a significant effect on the teachers’ AU of using microgames. This was because the games were developed in a simple and easy-to-use pattern, with very friendly and suitable features for use by the teachers without the ability to integrate institutional technology. In this case, the mathematics teachers showed that the utilization of microgames did not require TPACK ability, as perceived usefulness and ease-of-use importantly influenced their behavioral intentions toward using the learning model. Based on these results, the games were directly used in educational activities and not necessarily influenced by TPACK.

## Implications

This study theoretically extends the literature on microgame adoption in mathematics education, where the program was found to help improve teaching performance. However, the use of these games may not completely effective in improving the quality of teaching and learning activities in schools. The result possibly had a good contribution in developing countries, where it is a pioneer in analyzing behavioral intention and actual usage of microgames. The development of the technology acceptance model (TAM) was also well-established by adopting relevant factors, including PU, PEU, SN, and FC. This was in line with the model extension, which involved the addition of self-efficacy, TA, and TPACK. In previous reports, these factors were observed to have a relationship with the behavioral intention and actual use of new technology (Fang and Liu, 2017; Gellerstedt et al., 2018; Adov et al., 2020; Naveed et al., 2020; Fussell and Truong, 2022). The selection and extension of these predictors were also based on the integration of social and technical abilities, knowledge, and psychographics into a perfect model. However, a correlation was observed regarding online learning and video-based instruction when other educational studies indicated the relationship between self-efficacy, TPACK, and PU on behavioral intention and actual use. Despite these conditions, microgames still exhibited some notable differences, where the aforementioned relationship was less significant from the perspective of the mathematics teacher. This revealed that TA and self-efficacy had a significantly positive and direct effect on the actual use of microgames.

Regarding practical implications, this study is the first analysis to predict the factors influencing the increased utilization of microgames at the secondary school level. This clarified that decision-makers had a better understanding of considerable predictors when attempting to futuristically increase the use of these programs. The results may also help lecturers and software developers to better understand the factors to be considered, replaced, and modified, according to the wishes of the educational sector. Besides considering the programmatic quality to improve student learning outcomes, developers should assume more about the easy-to-use aspects of the technology, to motivate the math teachers towards using microgames. Furthermore, principals and curriculum departments need to plan and promote the awareness of these teachers on the importance and benefits of integrating institutional technology and appropriately using the games, respectively. The results also suggested the consideration of the school facility conditions and the provision of an adequate support system. In this condition, the school facilities supporting the integration of microgames into teaching and learning activities are further observed as important considerable factors.

## Conclusion

Microgames are one of the technology-based learning media with many benefits for students, regarding the improvement of quality teaching and learning activities in mathematics. To increase the use of these educational microgames by teachers, local governments, and schools, it was important to know the factors influencing the behavioral intention and actual use of the respective users. Based on the results, the essential BI and AU of microgames were investigated and predicted, leading to the modification of the TAM model by adding self-efficacy, TPACK, and TA. These factors were subsequently predicted to have a direct effect on behavioral intention and actual use. From the problem formulation, the results obtained exhibited the following, (1) perceived easy to use and Social Effect were the factors affecting the BI of teachers to use microgames, (2) TA, BI, and FC were the factors impacting the significant direct effect on the actual use of the games, (3) Social Effect had the largest significant effect on BI, and (4) Behavioral Intention had the greatest effect on the actual use of microgames. The theoretical and practical implications of this report also provided knowledge for the local governments and schools predicted to increase the implementation and adoption of microgames in mathematics lessons.

## Limitations and future works

Several significant limitations were still observed despite the positive results obtained, including (1) The development and use of microgames in mathematics are relatively new, indicating that the model only explained ~43% of the actual usage influential factors. This clarified that there were still many factors to be investigated besides those that were found, (2) This study is limited to only West Java, Indonesia, where all the respondents had received microgames training for not <1 year. Therefore, the number of respondents needs to be increased in subsequent analysis, (3) The technology acceptance model (TAM) was only considered, as many better techniques were still available for predicting the intention to use new technology, including UTAUT-2, and (4) The microgames were only developed using VBA Microsoft Excel, PowerPoint, and MIT Scratch, which were simple and easily modified by the teachers. Therefore, these games need to be developed with many software and other professional programs in subsequent future reports, such as the GeoGebra applet and python. Comparative analysis should also be futuristically performed, to know whether these kinds of microgames have similar results as this study.

## Data availability statement

The original contributions presented in the study are included in the article/Supplementary material, further inquiries can be directed to the corresponding authors.

## Ethics statement

The studies involving human participants were reviewed and approved by Faculty of Mathematics and Science Education, IKIP Siliwangi, Bandung, Indonesia. The patients/participants provided their written informed consent to participate in this study.

## Author contributions

All authors listed have made a substantial, direct, and intellectual contribution to the work and approved it for publication.

## Funding

This research was funded by the International Joint Research Project of Faculty of Education, Beijing Normal University.

## Conflict of interest

The authors declare that the research was conducted in the absence of any commercial or financial relationships that could be construed as a potential conflict of interest.

## Publisher’s note

All claims expressed in this article are solely those of the authors and do not necessarily represent those of their affiliated organizations, or those of the publisher, the editors and the reviewers. Any product that may be evaluated in this article, or claim that may be made by its manufacturer, is not guaranteed or endorsed by the publisher.

## Supplementary material

The Supplementary material for this article can be found online at: https://www.frontiersin.org/articles/10.3389/fpsyg.2022.952549/full#supplementary-material
