# Supplementary material for: Factors influencing microgame adoption among secondary school mathematics teachers supported by structural equation modelling-based research
Source: Front Psychol. 2022 Sep 8;13:952549. doi: 10.3389/fpsyg.2022.952549 (PMC9493482; doi:10.3389/fpsyg.2022.952549)
Supplement: Supplementary file 1 [file Table_1.docx]

# Appendix 1. Detailed questionnaire items

| **Variable construct** | **Question** |
| --- | --- |
| Perceived usefulness | I think microgames are very useful in teaching and learning mathematics |
|  | I think using microgames can increase the effectiveness of learning mathematics |
|  | Using microgames is easy to improve student learning outcomes |
|  |  |
| Perceived ease of use | Learning to use microgames to teach math is very easy |
|  | I feel that microgames are very flexible and practical to use for teaching mathematics |
|  | Overall, I find microgames easy to use |
|  |  |
| Subjective norm | I use microgames because the school told me to use VBA based microgames |
|  | I use microgames because other friends also use microgames to teach math |
|  |  |
| Facilitating conditions | The school provides computers, tablets, or laptop devices to use VBA-based microgames |
|  | Some people will help me if I do not know how to use microgames in math lessons |
|  |  |
| Teacher Attitudes towards microgames | Using microgames to teach math is a good idea |
|  | I feel very happy when I can use microgames when teaching math |
|  | Microgames make maths lessons more interesting |
|  | My attitude towards microgames overall is very positive |
|  |  |
| Self-efficacy | I am confident in using microgames to teach math even though people around me do not use microgames |
|  | I am confident in using microgames to teach math even though I have never used microgames before |
|  | I am confident in using microgames to teach math even though I have to make the microgames myself |
|  |  |
| TPACK | I can use microgames to increase students' interest in learning and motivation in math lessons |
|  | I can combine my teaching methods with microgames to teach math material |
|  | I can share learning models and microgames to teach mathematical topics |
|  |  |
| Behavior intention | I will continue to use microgames in math lessons |
|  | I will use microgame again next school year |
|  | I will be intense to use microgame in the future. |
|  |  |
| Actual use of microgames | I use microgames on every math topic |
|  | I recommend microgame to friends |
|  | I often give microgames to students when teaching math |
